# Dietary patterns and associated factors of schooling Ghanaian adolescents

**DOI:** 10.1186/s41043-019-0162-8

**Published:** 2019-02-06

**Authors:** Abdul-Razak Abizari, Zakari Ali

**Affiliations:** Department of Nutritional Sciences, School of Allied Health Sciences, University for Development Studies, P O Box 1883, Tamale, Ghana

**Keywords:** Adolescents, Dietary pattern, School children, Anthropometric status, Ghana

## Abstract

**Background:**

Assessment of single nutrients or foods does not normally reflect the diet of population groups. Dietary pattern analyses are useful in understanding the overall diet and its relationship with disease conditions. The objective of the present study was to determine the dietary patterns and associated factors among schooling adolescents in Northern Ghana.

**Methods:**

A cross-sectional study involving 366 pupils in 10 junior high schools in the Tamale metropolis was conducted. A Food Frequency Questionnaire (FFQ) which consisted of 60 commonly consumed foods was used to assess pupils’ 7-day intake. Foods grouped (14) from FFQ data based on shared nutritional value were used to identify dietary patterns using principal component analysis (PCA). Bivariate and multivariate logistic regression analyses were used to determine the association between identified patterns and sociodemographic, anthropometric status, and household characteristics of pupils.

**Results:**

Half of the pupils were female (50.3%) and average age was 15.6 ± 2.0 years. PCA identified two dietary patterns which in total explained 49.7% of the variability of the diet of pupils. The patterns were sweet tooth pattern (STP) with high factor loadings for sugar sweetened snacks, energy and soft drinks, sweets, tea and coffee, and milk and milk products, and a traditional pattern (TP) which showed high factor loadings for cereals and grains, local beverages, nuts, seeds and legumes, vegetables, and fish and seafood. Logistic regression showed that pupils who lived with their parents [AOR = 1.95; 95% CI (1.1–3.4); *p* = 0.019], those who went to school with pocket money [AOR = 4.73; 95% CI (1.5–15.0); *p* = 0.008], and those who lived in the wealthiest homes [AOR = 3.4; 95% CI (1.6–7.5); *p* = 0.002)] had higher odds of following the STP. The TP was associated with high dietary diversity (*p* = 0.035) and household wealth [AOR = 3.518; 95% CI (1.763–7.017); *p* < 0.001)]. None of the patterns was associated with anthropometric status of pupils.

**Conclusion:**

Adolescents in the present study followed a sweet tooth or a traditional diet pattern which associated more with household- and individual-level factors but not anthropometric status.

**Electronic supplementary material:**

The online version of this article (10.1186/s41043-019-0162-8) contains supplementary material, which is available to authorized users.

## Background

Increasing urbanization, changing diets, and decreasing physical activity levels are core indicators of the nutrition transition experienced by nations worldwide [1]. The nutrition transition is increasingly driving the world’s population towards an obese one, which is burdened with chronic diseases [1]. Despite an existing communicable disease burden and malnutrition in low- and middle-income countries [2], the developing world is not spared of non-communicable diseases [3] previously thought of as a problem of the developed world. The nutrition transition is thought to be a major driver of the rise in non-communicable diseases in developing countries [3, 4].

Ghana is among a few sub-Saharan African countries at a later stage of the nutrition transition where diet changes are already affecting the health of majority of the population [5]. Changing dietary intake and habits among the Ghanaian population was noticed earlier in the 1990s and described [6]. These changing patterns relate more to giving away traditional foods (which are mostly plant based and less processed) towards convenient foods (including fast and processed foods).

There is evidence of coexisting burden of underweight and overweight and obesity in schooling adolescents in resource poor settings including Ghana [7]. While there has been 18.3 percentage points decrease in underweight among schooling adolescents in Ghana between 2007 and 2015, there has been an increase in overweight and obesity from 8.7% in 2007 to 13% in 2015 [7, 8]. Associations between sociodemographic, household factors [9, 10] and anthropometric status [11], and dietary patterns of adolescents have been reported in different settings.

Dietary intake of adolescents in developing countries including Ghana [12] is a concern as traditional diets (predominantly cereal and tuber based, fresh fruits and vegetables, and foods low in fat) are gradually giving way to more Westernized diets which lack diversity and are high in calorie-rich processed foods [13]. As adolescents spend most time in school coupled with the autonomy to make food choices on their own while in school, the school environment is an important factor in shaping dietary patterns. It may present an opportunity to encourage physical activity and steer dietary intake of adolescents towards healthier options [14] or lead to poor dietary habits [15]. Identifying patterns of dietary intake therefore, could be a reliable way to understand the dietary behavior of adolescents and inform interventions for improved dietary intake.

However, most studies in the past have assessed only single nutrients or single food intake which do not normally reflect the overall diet of population groups. Available data on food intake of adolescents and school children in Ghana have also focused on individual food items and nutrients [12, 16]. Dietary pattern analysis has emerged as a useful epidemiological approach to assessing the overall diet and its relation with disease conditions [17]. Adolescent dietary patterns have been useful in understanding long-term adiposity [18] and occurrence of chronic diseases [19, 20]. Dietary patterns also have an added advantage of being easily understood and used by the general population. The objective of the present study was to determine the dietary patterns and associated factors among schooling adolescents in Northern Ghana.

## Methods

### Study design and area

The present study uses baseline data from the Ramadan cohort study conducted in junior high schools (JHSs) in May 2017 in Ghana. The baseline data was collected through a cross-sectional survey among schooling adolescents before Ramadan fasting. Details of the Ramadan cohort study have been described and published elsewhere [21]. There are 15 educational circuits in the metropolis with a total of 72 JHSs. The metropolis has a youthful population where about 36.4% are aged younger than 15 years. For the school going age (older than 3 years), enrolment in primary schools is about 60,000. The JHS also enroll 26,936 of these pupils [22].

### Study population and sampling

Adolescents (aged 10–19 years inclusive) in junior high school (JHS) were the target population for this survey. We used simple random sampling technique to select half (7) of the educational circuits in the Metropolis. Junior high schools in the selected circuits were pooled together from which 10 were randomly selected for the present study. Three hundred and sixty-six pupils were selected from the 10 schools using probability proportional to size methodology. The required sample from each school was selected from a list of eligible participants by simple random sampling technique using excel-generated random numbers. Participation was voluntary, no monetary incentives were given, and selected pupils also gave signed informed consent before data collection.

### Data collection

Data was collected using pre-tested semi-structured questionnaire. The questionnaire elicited responses on socio-demographic characteristics of pupils such as sex, age, ethnicity, class, means to school, pocket money to school (pupils who usually go to school with pocket money for at least 3 out of 5 school days), and living with parents (defined as pupils who live with their biological parent(s)). The questionnaire also elicited responses on household characteristics such as parental educational level and occupation. Educational level of parents was assessed as the highest level of education parents completed, and parental occupation was assessed as the primary occupation of parents. We also assessed household possession of some durable household items. Data collection staff were first-degree nutritionists and received training on questionnaire administration, dietary assessment, and anthropometric measurements before data collection. Each school also had a field supervisor who provided onsite checks on questionnaire, and incomplete questionnaire and measurement errors were corrected the same day.

### Dietary assessment

We assessed dietary diversity using a qualitative 24-h recall. We used the Food and Agricultural Organization’s (FAO) food groups for dietary diversity and their standard procedure for assessing individual dietary diversity [23]. Foods consumed both at home and school during the previous day were recalled by pupils. Based on 14 food groups, we calculated the dietary diversity score (DDS) for each pupil which was a count of food groups pupils consumed during the previous day preceding the survey. Pupils had a score of 0 if none of the food groups was consumed and 14 if all food groups were consumed. The 14 food groups used in the DDS calculation were cereals; white roots and tubers; vitamin A rich vegetables and tubers; dark green leafy vegetables; other vegetables; vitamin A-rich fruits; other fruits; organ meat; flesh meats, eggs; fish and seafood; legumes, nuts and seeds; milk and milk products; and oils and fats (Additional file 1).

We also used a 7-day Food Frequency Questionnaire (FFQ) consisting of 60 food items commonly consumed in Ghana (see Additional file 2). The foods in this questionnaire were similar to those used previously in the northern region [24]. Pupils recalled how often they have had, on average, a particular food item in the week preceding the assessment. Consumption scores ranged from 0 (when they had never or hardly ever taken a particular food for the past week) to 7 (if they had a particular food for more than 6 days in the past 1 week). The foods were regrouped into 14 sub-groups for use in principal component analysis (PCA) by adding scores of foods belonging to similar food groups (foods with shared nutritional value). The 14 food groups include cereals and grains; tubers and plantain; local beverages with added sugar; tea and coffee; sugared snacks; sweets; meats, poultry and eggs; fish and seafood; milk and milk products; nuts, seeds and legumes; fruits and fruit juices; vegetables; energy and soft drinks; fats, oils and fat-based foods.

### Anthropometric status assessment

Weight and height measurements were taken following World Health Organization (WHO) standard procedure [25]. Weight was measured using an electronic weighing scale (seca 874) to the nearest 0.1 kg. Height was measured with seca stadiometer to the nearest 0.1 cm. Data collection staff received training before the assessment. Age, sex, height, and weight data were used in WHO AnthroPlus software to generate *Z*-scores on BMI-for-age and sex (BMIA). The *Z*-scores were categorized into normal (BMIA ≤ 1SD), overweight (BMIA >1SD), and obese (BMIA > 2SD) [26].

### Data analysis

Data analysis was performed using SPSS for Windows version 20 (IBM Inc.). Categorical variables have been presented as frequencies and percentages while means and standard deviations are used for continuous variables. The reliability of items in the questionnaire for PCA was checked using Cronbach’s alpha test statistic. Data collected using FFQ was used in PCA to assess dietary patterns of pupils. The 14 food groups were used to find foods that correlate highly to describe particular dietary patterns in PCA. Orthogonal rotation method using Varimax was used to maximize loadings of variables on extracted factors while minimizing loading on other factors; ensuring easy interpretation of the results. We used Kaiser’s stopping rule which considers factors with eigenvalues greater than 1.0 to be retained [27]. We also examined scree plots to confirm adequacy of number of factors retained in the analysis. Food groups that had factor loadings ≥ 0.4 were considered as making significant contribution [28] to a particular pattern. Sample adequacy of data suitable for PCA was assessed by Kaiser-Meyer-Olken (KMO) measure test which showed acceptable cut-off (> 0.9). Bartlett’s test of sphericity (BTS) performed on the data set did not show evidence of identity of correlation matrix; therefore, the data set was considered appropriate for PCA. Dietary patterns derived from PCA were labeled appropriately based on food items that correlated highly to account for variation in the diet. Factor scores of the identified patterns were used for further analysis. Calculation of pattern-specific factor scores were obtained as the sum of factor loading coefficients and the standardized consumption of the foods related to the dietary pattern. Factor scores were divided into four quartiles on the basis of their contribution to each pattern and assuming an increase from Q1 to Q4 [18, 29]. Q1 and Q2 were combined to represent low followers while Q3 and Q4 were combined to represent high followers of identified patterns. Household wealth status was assessed from possession of 14 durable items including radio, color/black TV, satellite dish, sewing machine, mattress, refrigerator, DVD/VCD, computer, electric fan, mobile telephone, bicycle, motorcycle/tricycle, animal-drawn cart, and car/truck. Based on these, the wealth index was determined using PCA and categorized into quintiles (poorest, poor, medium, wealthy, and wealthiest) [30, 31]. Factors associated with the identified patterns were determined using chi-square test at the bivariate level. Factors with *p* ≤ 2.0 at the bivariate analysis were included in a multivariable logistic regression model. Statistical significance was set at *p* < 0.05 for all analyses.

## Results

### Background characteristics of participating pupils

Half of the pupils were female (50.3%), were aged at least 15 years (52.2%), and were in JHS 1 (51.4%). More than 8 in 10 of adolescents in this sample had normal anthropometric status (88%), while only 6% were either overweight or obese. The pupils belonged to the Dagomba ethnic group (87.2%), lived with their parents (78.4%) in extended family homes (58.5%), and went to school with pocket money (92.6%). More than half (53.3%) of the fathers had no formal education and engaged in farming activities (32.8%). Most mothers (70%) had no formal education and were mostly traders (71.3%). Most of the pupils (59%) were from households of at least a medium wealth classification (Table 1).

**Table 1:** Background characteristics of participating pupils (*n* = 366)

Characteristic	Frequency	Percentage
Sex		
Male	182	49.7
Female	184	50.3
Age (years)		
≤ 15	175	47.8
> 15	191	52.2
Class of pupil		
JHS 1	188	51.4
JHS 2	178	48.6
Anthropometric status (BMI-for-age)		
Underweight	23	6.3
Normal	321	87.7
Overweight/obese	22	6.0
Ethnicity		
Dagomba	319	87.2
Gonja	26	7.1
Others	21	5.7
Live with parents?		
Yes	287	78.4
Family type		
Nuclear	152	41.5
Extended	214	58.5
Go to school with pocket money		
Yes	339	92.6
Father’s education		
None	195	53.3
Primary/junior high school	98	26.8
Senior high school/tertiary	73	19.9
Father’s occupation		
Farmer	120	32.8
Trader	74	20.2
Civil servant	33	9.0
Others	139	38.0
Mother’s education		
None	256	69.9
Primary/junior high school	83	22.7
Senior high school/tertiary	27	7.4
Mother’s occupation		
Farmer	35	9.6
Trader	261	71.3
Civil servant	6	1.6
Others	64	17.5
Household wealth quintile		
Poorest	74	20.2
Poor	76	20.8
Medium	70	19.1
Wealthy	73	19.9
Wealthiest	73	19.9

### Dietary patterns of participating pupils

KMO (0.917) and BTS (approx chi (1836.92) *p* < 0.001) showed that the data was adequate for PCA. The items also showed high reliability (Cronbach’s alpha = 0.869). The items had communality values well above 0.3 indicative of the appropriateness of the number of components retained. Two dietary patterns were identified which together explained 49.7% of dietary intake of pupils. The components were labeled *sweet tooth pattern* (STP) and *traditional pattern* (TP)*.* The STP, which explained most (32.5%) of variance, was characterized by intake of sugar sweetened snacks, energy and soft drinks, sweets (chewing gums and toffees), tea and coffee, milk and milk products, and fats and high fat-based foods. The TP which explained the rest of the variance (17.2%) was characterized by consumption of cereals and grains, local beverages, nuts, seeds and legumes, vegetables, and fish and seafood (Table 2).

**Table 2:** Dietary patterns of participating pupils

Food items	Dietary patterns with factor loadings		Communalities
	Sweet tooth	Traditional	
Milk and milk products	*.801*	.087	.648
Fruits and fruit juice	*.756*	.251	.635
Meat, poultry, and eggs	*.749*	.153	.584
Energy and soft drinks	*.684*	.206	.510
Tubers and plantain	*.636*	.243	.464
Sugared snacks	*.626*	.293	.478
Sweets	*.605*	.307	.460
Fats, oils, and fat-based foods	*.587*	.377	.486
Tea and coffee	*.574*	.056	.332
Fish and seafood	− .077	*.747*	.564
Nuts, seeds, and legumes	.287	*.740*	.630
Local sugared beverages	*.420*	*.540*	.467
Vegetables	.203	*.524*	.316
Cereals and grains	*.411*	*.457*	.378
*% of variance explained*	32.516	17.156	
*% of accumulated variance explained*	49.672		

Extraction method, principal component analysis. Rotation method, varimax with Kaiser normalization. Italic factor loadings are items contributing significantly to each PCA component

### Determinants of dietary patterns of participating pupils

The bivariate chi-square results show that, sex of pupil is not associated with either of the dietary patterns (*p* > 0.05). Even though older pupils were more likely to have high scores of the diet patterns, the difference was not significant. Pupils who lived with their parents were more likely to have high STP (53.3% vs 38.0%, *p* = 0.016). Living with parents was not associated with the TP (*p* = 0.899). Pupils who went to school with pocket money were more likely to practice the STP (*p* < 0.001) but not the TP (*p* = 0.842). Dietary diversity was significantly associated with the TP (*p* = 0.035) but not the STP (*p* = 0.074). For example, most (52.0%) pupils who consumed from at least four food groups had high scores on the STP compared with those who did not (35.0%). Anthropometric status of pupils was not significantly associated with the two diet patterns (*p* > 0.05). However, some other marked differences were obvious. Father’s educational level (*p* = 0.033) and employment type (*p* = 0.006) were associated with STP but not the TP (*p* > 0.05). Pupils whose fathers had higher education of at least senior high school and worked in civil service had higher scores on the STP. Mothers’ educational level and employment type were not significantly associated with both dietary patterns. Household wealth status was associated with the STP (*p* < 0.001) as well as the TP (*p* = 0.003). There was an observed increasing scores with increasing household wealth for both dietary patterns where pupils from the wealthiest households scored higher (Table 3).

**Table 3:** Bivariate analysis of the predictors of dietary patterns of participating pupils

Predictor	Dietary pattern					
	Sweet tooth			Traditional		
	Low (183) *n* (%)	High (183) *n* (%)	*p* value	Low (183) *n* (%)	High (183) *n* (%)	*p* value
Sex			0.210			0.676
Male	85 (46.7)	97 (53.3)		89 (48.9)	93 (51.1)	
Female	98 (53.3)	86 (46.7)		94 (51.1)	90 (48.9)	
Age (years)			0.917			0.464
≤ 15	88 (50.3)	87 (49.7)		91 (52.0)	84 (48.0)	
> 15	95 (49.7)	96 (50.3)		92 (48.2)	99 (51.8)	
Live with parents			*0.016*			0.899
Yes	134 (46.7)	153 (53.3)		144 (50.2)	143 (49.8)	
No	49 (62.0)	30 (38.0)		39 (49.4)	40 (50.6)	
Pocket money to school			*< 0.001*			0.842
Yes	160 (47.2)	179 (52.8)		170 (50.1)	169 (49.9)	
No	23 (85.2)	4 (14.8)		13 (48.1)	14 (51.9)	
Dietary diversity (food groups)			0.074			*0.035*
< 5	27 (62.8)	16 (37.2)		28 (65.1)	15 (34.9)	
≥ 5	156 (48.3)	167 (51.7)		155 (48.0)	168 (52.0)	
Nutritional status			0.320			0.358
Underweight	10 (43.5)	13 (56.5)		10 (43.5)	13 (56.5)	
Normal	165 (51.4)	156 (48.6)		159 (49.5)	162 (50.5)	
Overweight/obese	8 (36.4)	14 (63.6)		14 (63.6)	8 (36.4)	
Father’s educational level			*0.033*			0.858
None	107 (54.9)	88 (45.1)		95 (48.7)	100 (51.3)	
Primary/junior high school	49 (50.0)	49 (50.0)		51 (52.0)	47 (48.0)	
Senior high/tertiary	27 (37.0)	46 (63.0)		37 (50.7)	36 (49.3)	
Father’s occupation			*0.006*			0.392
Agriculture/farming	66 (55.0)	54 (45.0)		64 (53.3)	56 (46.7)	
Trader	39 (52.7)	35 (47.3)		32 (43.2)	42 (56.8)	
Civil servant	7 (21.2)	26 (78.8)		14 (42.4)	19 (57.6)	
Others	71 (51.1)	68 (48.9)		73 (52.5)	66 (47.5)	
Mother’s educational level			0.392			0.592
None	134 (52.3)	122 (47.7)		127 (49.6)	129 (50.4)	
Primary/junior high school	37 (44.6)	46 (55.4)		40 (59.3)	43 (51.8)	
Senior high/tertiary	12 (44.4)	15 (55.6)		16 (59.3)	11 (40.7)	
Mother’s occupation			0.170			0.859
Agriculture/farming	23 (65.7)	12 (34.3)		17 (48.6)	18 (51.4)	
Trader	124 (47.5)	137 (52.5)		131 (50.2)	130 (49.8)	
Civil servant	2 (33.3)	4 (66.7)		2 (33.3)	4 (66.7)	
Others	34 (53.1)	30 (46.9)		33 (51.6)	31 (48.4)	
Wealth quintile			*< 0.001*			*0.003*
Poorest	48 (64.9)	26 (35.1)		47 (63.5)	27 (36.5)	
Poor	48 (63.2)	28 (36.8)		38 (50.0)	38 (50.0)	
Medium	35 (50.0)	35 (50.0)		38 (54.3)	32 (45.7)	
Wealthy	32 (43.8)	41 (56.2)		37 (50.7)	36 (49.3)	
Wealthiest	20 (27.4)	53 (72.6)		23 (31.5)	50 (68.5)	

Predictors with *p* values in italics are statistically significant

Multivariable logistic regression analyses showed that living with parents, going to school with pocket money, and household wealth status was associated with STP. Pupils who lived with their parents had higher odds (2.0) of having a high STP [AOR = 1.95; 95% CI (1.1–3.4); *p* = 0.019]. Those who went to school with pocket money were 4.7 times more likely to have high STP [AOR = 4.7; 95% CI (1.5–15.0); *p* = 0.008]. Compared to pupils who lived in the poorest homes, those who lived in the wealthiest homes were almost 3.4 times more likely to have an STP [AOR = 3.4; 95% CI (1.6–7.5); *p* = 0.002)].

Only household wealth status was associated with the TP in regression analysis. The results show that pupils from the wealthiest homes were 3.5 times more likely to follow TP compared with those from the poorest homes [AOR = 3.5; 95% CI (1.8–7.0); *p* < 0.001) (Table 4).

**Table 4:** Logistic regression analysis of the determinants of high STP and TP among pupils

	AOR	*p* value	95% CI for AOR	
			Lower	Upper
Sweet tooth pattern (STP)				
Sex				
Male	1.12	0.644	0.70	1.80
Female	1			
Live with parents?				
Yes	1.95	*0.019*	1.11	3.40
No	1			
Pocket money to school?				
Yes	4.73	*0.008*	1.49	14.99
No	1			
Dietary diversity (food groups)				
Less than 5	0.76	0.460	0.37	1.57
Above 5	1			
Father’s educational level		0.803		
None	0.85	0.650	0.42	1.72
Primary/junior high school	0.78	0.510	0.37	1.63
Senior high/tertiary	1			
Mother’s occupation		0.972		
Agriculture/farming	0.84	0.728	0.32	2.23
Trader	0.97	0.925	0.53	1.77
Civil servant	0.71	0.733	0.10	5.19
Others	1			
Father’s occupation		0.173		
Agriculture/farming	1.09	0.768	0.61	1.96
Trader	0.83	0.563	0.45	1.54
Civil servant	3.04	*0.044*	1.03	8.98
Others	1			
Household wealth		0.004		
Wealthiest	3.40	*0.002*	1.55	7.46
Wealthy	1.59	0.218	0.76	3.35
Medium	1.35	0.430	0.64	2.86
Poor	0.86	0.685	0.42	1.77
Poorest	1			
Traditional pattern (TP)				
Household wealth		0.008		
Wealthiest	3.52	*< 0.001*	1.76	7.02
Wealthy	1.62	0.156	0.83	3.14
Medium	1.38	0.348	0.70	2.71
Poor	1.68	0.120	0.87	3.25
Poorest	1			
Dietary diversity (food groups)				
Less than 5	0.57	0.103	0.29	1.12
Above 5	1			

Predictors with *p* values in italics are statistically significant

## Discussion

The present study assessed dietary patterns of adolescents in junior high schools in predominantly urban areas of the Tamale metropolis and factors associated with the patterns. Two distinct dietary patterns were identified among the pupils; sweet tooth pattern (STP) and traditional pattern (TP). The STP was characterized by intake of sugar sweetened snacks, energy and soft drinks, sweets (chewing gums and toffees), tea and coffee, milk and milk products, and fats and high fat-based foods. The TP was characterized by consumption of cereals and grains, local beverages, nuts, seeds and legumes, vegetables, and fish and seafood. STP was associated more with household socio-economic factors including household wealth, pupil having pocket money to school, and living with parents. The TP was also associated with household wealth and dietary diversity.

The dietary patterns identified in this study are similar to those identified among school-age children in a metropolitan area of Southern Ghana by Alangea et al. [32]. Alangea et al. identified four dietary patterns, the first of which had food characteristic of the STP in this study. However, the traditional pattern as identified in this study could be traced to three separate dietary patterns: starchy root staple and vegetables, cereal-grain staples and poultry, and fish and sea foods.

Dietary patterns in the present study were not associated with overweight or obesity among adolescents. Even though overweight or obese adolescents scored relatively higher on the STP while those with lower BMI scored relatively higher on the TP, these were not significant. The sweet tooth pattern which had foods typically of a modern, an affluent, or a westernized nature have been identified in previous studies among children and adolescents including in China [18], Australia [33], The Netherlands [34], UK [35], Germany [36], and in Ghana [32]. Foods in this pattern are largely energy-dense and have previously been linked with overweight or obesity in children and adolescents elsewhere [18, 32, 37]. However, this has not been the same in all studies that assessed dietary patterns of adolescents. A lack of association between an energy-dense pattern and dietary patterns in general and overweight or obesity among adolescents has been reported in the literature. For example, Shi et al. [33] identified a “processed food” pattern characterized by consumption of processed meats, snacks, and sugary foods among Australian children which was not associated with obesity. Cutler et al. [11] could not find intuitive associations between dietary patterns of US adolescents and weight status. An “unhealthy pattern” characterized by snack and puddings intake identified by Craig et al. [35] was not also associated with overweight or obesity among Scottish school-age children. Further, no significant associations were found between adolescent dietary patterns and overweight in German adolescents [36]. The lack of association is contrary to our expectation that adolescents who follow the STP would more likely be overweight or obese as they may be taking more energy. The reasons for the lack of association between a diet pattern high in energy and sweets and overweight or obesity among adolescents are unclear. However, few explanations are plausible. In this physiological group, diet might not be the only important determinant of over-nutrition; effects of physical activity may equally be important [38]. In addition, important confounding may exist when overweight adolescents may have consciously attempted to lose weight [39]. In our setting and as with other studies using FFQ, social desirability bias in dietary assessments may be unavoidable [40, 41], resulting in over-reporting of some foods, especially with foods characteristic of the STP which are more likely to be seen as affluent foods among Ghanaians. On the other hand, under-reporting of fatty foods and foods high in energy may be high among the obese [42]. However, the former is more likely among our participants and may have led to misclassification of some adolescents into the dietary patterns. Further, as portion sizes were not estimated in the present study, similar intake frequencies may not necessarily mean similar exposure levels as portions may differ between individuals. Moreover, dietary patterns identified with PCA are rarely entirely made of foods that promote or are harmful to health. The effects of a particular pattern on health will principally depend on the individual foods that make up the pattern and may explain the inconsistencies with studies. The low prevalence of overweight or obesity in this study could also lead to low statistical power to detect a significant association.

The association between the dietary patterns and socio-economic factors are consistent with earlier findings. In our sample, higher socio-economic status was associated with STP similar to a recent review of the literature which concluded that high socio-economic status was associated with unhealthy eating patterns in developing country setting but with healthy eating patterns among adolescents in developed countries [43]. Our findings therefore, do not agree with McNaughton et al. [10] who did not find association between dietary patterns and socio-economic status. Adolescents who live with their parents may have advantages with household food shares including money to school. An earlier study in northern region of Ghana reported disproportionate food shares between children of household heads and other children within the same household [44]. Pupils from high wealth homes may go to school with pocket money and therefore, are able to purchase sweets, snacks, and soft drinks. The association between living with parents and pocket money and the STP is therefore understandable. Following from these, it may be important for adolescents to receive nutrition education and guidance regarding food choices especially while in school where they make independent food choices. Equally important is the need to make the food environment of schools healthier to influence healthy choices.

The high dietary diversity descriptive of the TP in bivariate analysis but not the STP may be explained by the food characteristic of the patterns. Food characteristic of the STP such as sweets, energy and soft drinks, teas, and coffees are typically not included in the calculation of dietary diversity. However, foods characteristic of the TP such as cereals and grains, nuts, seeds and legumes, fruits, vegetables, and fish and sea foods are included in the calculation of dietary diversity [23, 45]. As increased dietary diversity may mean nutrient adequacy among adolescents [46, 47], it may be reasonable to promote a TP way of eating among adolescents.

The interpretation of the findings of this study should be made with some limitations in mind. The present study used a cross-sectional design and hence no causal links can be implied. The use of a qualitative approach to dietary intake assessment may not reveal the actual exposure level. The use of FFQ which rely on respondent memory over along exposure period may introduce recall bias which can affect our findings. However, dietary intake assessments using FFQ have been shown to be reliable in revealing usual intake which is important in the present study [40, 41]. In spite of these limitations, our data reveal important dietary patterns and associated factors among urban adolescents in the Tamale metropolis of northern Ghana.

## Conclusion

Schooling Ghanaian adolescents in the present study followed a sweet tooth or traditional diet pattern. Household wealth, living with parents, and going to school with pocket money were associated with the sweet tooth pattern. The traditional pattern was associated with household wealth. Identified patterns were not associated with anthropometric status.

## Additional files

Additional file 1: Food groups based on foods in the FFQ and used for PCA. (DOCX 14 kb)

Additional file 2: Seven day Food Frequency Questionnaire. (DOCX 16 kb)

## Abbreviations

AOR: Adjusted odds ratio
FFQ: Food Frequency Questionnaire
JHS: Junior high school
PCA: Principal component analysis
STP: Sweet tooth pattern
TP: Traditional pattern
