# Supplementary material for: Dietary patterns and associated factors of schooling Ghanaian adolescents
Source: J Health Popul Nutr. 2019 Feb 6;38:5. doi: 10.1186/s41043-019-0162-8 (PMC6364425; doi:10.1186/s41043-019-0162-8)
Supplement: Supplementary file 1 — Food groups based on foods in the FFQ and used for PCA. (DOCX 14 kb) [file 41043_2019_162_MOESM1_ESM.docx]

**Additional file 1: Food groups based on foods in the FFQ and used for PCA**

| **Food group** | **Food items** |
| --- | --- |
| Cereals and grains | Tuo zaafi, banku, kenkey, riceballs, pain rice, bread |
| Tubers and plantain | Fufu, yam, plantain, sweet potato |
| Meat, poultry and eggs | Meats (cow, goat, sheep, rabbit), poultry (chicken, duck, guinea fowl, turkey), eggs |
| Vegetables | Traditional vegetables, exotic vegetables |
| Fruits and fruit juice | Pineapple, watermelon, apple, orange, mango, banana, pear/avocado, pawpaw, shea fruit, date, berries, fruit juice |
| Milk and milk products | Milk, yoghurt, waagashie |
| Fish and seafood | Fish and seafood |
| Sugared snacks | Biscuits, chocolates |
| Tea and coffee | Tea, coffee |
| Sweets | Sweets (chewing gums, toffees), fanmilk, fanchoco |
| Nuts, seeds and legumes | Cow pea, peanut, pigeon pea, Bambara beans, soya beans |
| Local sugared beverages | Mashed kenkey, koko, zimkuom, sobolo, poha, bear |
| Energy and soft drinks | Energy drinks, soft drinks, malts |
| Fats, oils and fat based foods | Fats and oils, jollof, fried rice, masa, palmnut, coconut, |
