# Supplementary material for: Dietary patterns and associated factors of schooling Ghanaian adolescents
Source: J Health Popul Nutr. 2019 Feb 6;38:5. doi: 10.1186/s41043-019-0162-8 (PMC6364425; doi:10.1186/s41043-019-0162-8)
Supplement: Supplementary file 2 — Seven day Food Frequency Questionnaire. (DOCX 16 kb) [file 41043_2019_162_MOESM2_ESM.docx]

**Additional file 2: Seven day Food Frequency Questionnaire**

How often have you on average had the following in the past one week? (Please tick)

| Food item | Frequency | | | | | | |
| --- | --- | --- | --- | --- | --- | --- | --- |
|  | *>* 6 times a week | 5 times a week | 4 times a week | 3 times a week | 2 times a week | once a week | not at all |
| Tuo Zaafi (made from corn/millet flour) |  |  |  |  |  |  |  |
| Banku (made from fermented corn and/cassava dough) |  |  |  |  |  |  |  |
| Fufu (pounded yams/cassava) |  |  |  |  |  |  |  |
| Kenkey (made from fermented corn dough) |  |  |  |  |  |  |  |
| Rice and beans |  |  |  |  |  |  |  |
| Jollof (boiled rice with stew) |  |  |  |  |  |  |  |
| Fried rice |  |  |  |  |  |  |  |
| Rice balls |  |  |  |  |  |  |  |
| Plain rice |  |  |  |  |  |  |  |
| Yam (fried or boiled) |  |  |  |  |  |  |  |
| Sweet potato |  |  |  |  |  |  |  |
| Plantain |  |  |  |  |  |  |  |
| Bread |  |  |  |  |  |  |  |
| Biscuits |  |  |  |  |  |  |  |
| Mashed kenkey |  |  |  |  |  |  |  |
| Maasa (fried corn dough) |  |  |  |  |  |  |  |
| Porridge |  |  |  |  |  |  |  |
| Fula (made from millet dough) |  |  |  |  |  |  |  |
| Zimkuom (local drink made from corn/millet flour) |  |  |  |  |  |  |  |
| Sobolo (Roselle drink) |  |  |  |  |  |  |  |
| Meat (cow, goat, sheep) |  |  |  |  |  |  |  |
| Fish and seafood |  |  |  |  |  |  |  |
| Poultry |  |  |  |  |  |  |  |
| Egg |  |  |  |  |  |  |  |
| Milk |  |  |  |  |  |  |  |
| Yoghurt |  |  |  |  |  |  |  |
| Waagashie (local cheese) |  |  |  |  |  |  |  |
| Soya/soy kebab (soya beans) |  |  |  |  |  |  |  |
| Chocolate |  |  |  |  |  |  |  |
| Pineapple |  |  |  |  |  |  |  |
| Watermelon |  |  |  |  |  |  |  |
| Apple |  |  |  |  |  |  |  |
| Orange |  |  |  |  |  |  |  |
| Mango |  |  |  |  |  |  |  |
| Banana |  |  |  |  |  |  |  |
| Avocado /pear |  |  |  |  |  |  |  |
| Pawpaw |  |  |  |  |  |  |  |
| Shea fruit |  |  |  |  |  |  |  |
| Date (date fruit) |  |  |  |  |  |  |  |
| Berries (all kinds) |  |  |  |  |  |  |  |
| Soft drinks (coca cola, plastic coloured soft drinks) |  |  |  |  |  |  |  |
| Fan milk |  |  |  |  |  |  |  |
| Fan choco |  |  |  |  |  |  |  |
| Tea |  |  |  |  |  |  |  |
| Coffee |  |  |  |  |  |  |  |
| Poha (local tamarind drink) |  |  |  |  |  |  |  |
| Honey |  |  |  |  |  |  |  |
| Energy drink (5 star, Rush etc) |  |  |  |  |  |  |  |
| Malts (guinness, Rasta, Magic, etc) |  |  |  |  |  |  |  |
| Bear (local pepper drink) |  |  |  |  |  |  |  |
| Fruit juice (packed) |  |  |  |  |  |  |  |
| Traditional vegetables vegetables (bra leaves (*Hibiscus sabdariffa*), *ayoyo* leaves (*Corchorus olitorius*), *aleefu* (*Amarantus sp*.), tomato, red hot pepper, onions, baobab leaves (dry), okro (fresh fruits, fruit powder)) |  |  |  |  |  |  |  |
| Exotic vegetables (cabbage, lettuce, broccoli, carrots etc) |  |  |  |  |  |  |  |
| Sweets (toffee, gum etc) |  |  |  |  |  |  |  |
| Peanut (roasted, soup) |  |  |  |  |  |  |  |
| Cow pea (koose, boiled) |  |  |  |  |  |  |  |
| Adowa (pigeon pea) |  |  |  |  |  |  |  |
| Bambara beans |  |  |  |  |  |  |  |
| Palm nut |  |  |  |  |  |  |  |
| Coconut |  |  |  |  |  |  |  |
| Fats and oils (frytol, shea butter etc) |  |  |  |  |  |  |  |
